# Diabetes and vascular mild cognitive impairment among Chinese ≥50 years: A cross‐sectional study with 2020 participants

**DOI:** 10.1002/brb3.3477

**Published:** 2024-04-28

**Authors:** Yu Zhang, Wenna Liu, Chen Fu, Xuemei Liu, Xiaobing Hou, Huanmin Niu, Tao Li, Chunyan Guo, Aixun Li, Baoxin Chen, Xianglan Jin

**Affiliations:** ^1^ Department of Neurology Dongfang Hospital Beijing University of Chinese Medicine Beijing China; ^2^ Clinical Trial Institution Dongfang Hospital Beijing University of Chinese Medicine Beijing China; ^3^ Central Laboratory Dongfang Hospital Beijing University of Chinese Medicine Beijing China; ^4^ Department of Neurology Beijing First Hospital of Integrated Chinese and Western Medicine Beijing China; ^5^ Department of Gerontology Shanxi Traditional Chinese Medicinal Hospital Taiyuan China

**Keywords:** diabetes, education, protective factor, risk factor, vascular mild cognitive impairment

## Abstract

**Background:**

With the decline of cognitive function in vascular cognitive impairment, the burden on the family and society will increase. Therefore, early identification of vascular mild cognitive impairment (VaMCI) is crucial. The focus of early identification of VaMCI is on the attention of risk factors. Therefore, this study aimed to investigate the relationship between diabetes and VaMCI among the Chinese, hoping to predict the risk of VaMCI by diabetes and to move the identification of vascular cognitive impairment forward.

**Methods:**

We collected data from seven clinical centers and nine communities in China. All participants were over 50 years of age and had cognitive complaints. We collected basic information of the participants, and cognitive function was professionally assessed by the Montreal Cognitive Assessment scale. Finally, logistic regression analysis was used to analyze the correlation between each factor and VaMCI.

**Results:**

A total of 2020 participants were included, including 1140 participants with VaMCI and 880 participants with normal cognition. In univariate logistic regression analysis, age, heavy smoking, and diabetes had a positive correlation with VaMCI. At the same time, being married, high education, and light smoking had a negative correlation with VaMCI. After correction, only diabetes (OR = 1.04, 95% CI: 1.01–1.09, *p* = 0.05) had a positive correlation with VaMCI, and high education (OR = 0.60, 95% CI:.45–.81, *p* = 0.001) had a negative correlation with VaMCI.

**Conclusion:**

In our study, we found that diabetes had a positive correlation with VaMCI, and high education had a negative correlation with VaMCI. Therefore, early identification and timely intervention of diabetes may reduce the risk of VaMCI and achieve early prevention of VaMCI.

## Graphical abstract

In our study, we found that diabetes had a positive correlation with vascular mild cognitive impairment (VaMCI), and high education had a negative correlation with VaMCI. Therefore, early identification and timely intervention of diabetes may reduce the risk of VaMCI and achieve early prevention of VaMCI.

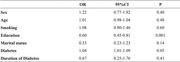

## 1 INTRODUCTION

Vascular cognitive impairment (VCI) is a clinical syndrome in which at least one cognitive domain is impaired by cerebrovascular disease and its risk factors. VCI covers two different stages of vascular mild cognitive impairment (VaMCI) and vascular dementia (VaD) and also includes cognitive impairment due to mixed pathologies such as comorbid Alzheimer's disease (Skrobot et al., 2018). As the gradual decline of cognitive function and the gradual loss of patients’ ability to care for themselves, VaMCI will progress to VaD. A 2‐year follow‐up study of VaMCI in Canada found that 40% of patients with VaMCI progressed to dementia, 52% remained in VaMCI (Hsiung et al., 2006). It meant that nearly half of the patients with VaMCI had a continuous decline in their cognitive function. Due to the gradual decline of cognitive function, patients with VaD will not be able to live independently, which will bring heavy caregiving and financial burdens to their families and society. Therefore, the identification and intervention of VaMCI is crucial. The early identification of VaMCI focuses on the attention and control of risk factors. In addition to a history of stroke such as cerebral infarction and cerebral hemorrhage, vascular risk factors, such as diabetes, hypertension, and hyperlipidemia, are important factors in the prevention and identification of VaMCI. It was well recognized that cerebrovascular lesions led to cognitive decline (Iadecola et al., 2019; Kalaria, 2017; Wolters & Ikram, 2019). Previous studies investigating diabetes and VaD correlation were also consistent with the results. Studies analyzing data from databases in the United States, Denmark, Switzerland, and the United Kingdom all found a positive correlation between diabetes and VaD (Celis‐Morales et al., 2022; Litkowski et al., 2023; Thomassen et al., 2020; Wang et al., 2023). However, the study aimed the association between diabetes and VaMCI was few. Therefore, this study aimed to investigate the association between diabetes and VaMCI among the Chinese people, hoping to predict the risk of VaMCI by diabetes and to move the identification of VCI forward. This study mainly aimed at middle‐aged and elderly people in China, conducted a cross‐sectional survey with a large sample, and analyzed the correlation between diabetes and diabetes duration and VaMCI.

## 2 MATERIALS AND METHODS

### 2.1 General data

The data were part of the Special Research Project for Traditional Chinese Medicine 2008, which aimed to identify risk factors for cognitive impairment in the elderly and to develop tools for rapid screening of cognitive impairment. The cluster sampling method was used. The project selected relatively stable and representative permanent residents aged 50 and above from seven centers and nine communities in the northern region as screening objects. The seven centers are all Grade 3A Hospital, including Dongfang Hospital of Beijing University of Chinese Medicine, Peking University People's Hospital, Wangjing Hospital of China Academy of Chinese Medicine Sciences, Second Teaching Hospital of Tianjin University of Traditional Chinese Medicine, The Affiliated Hospital of Shandong University of Traditional Chinese Medicine, The First Affiliated Hospital of Changchun University of Chinese Medicine, and Traditional Chinese Medicine Hospital of Hebei Medical University. The project was approved by the Medical Research Ethics Committee of Dongfang Hospital of Beijing University of Chinese Medicine. All subjects were from neurological outpatients, wards, and surrounding communities aged over 50 years in the above hospitals from June 2008 to April 2012. All participants signed the informed written consent. We developed a standardized observation form, and all observers passed a training and consistency test in neuropsychological scale assessment. Observers were responsible for one‐on‐one, face‐to‐face questioning and assessment based on the observation form. All the above, all the medical history and neuropsychological data were complete, and a total of 2020 objects were suitable for this study.

## 3 METHODS

### 3.1 Inclusion and exclusion criteria

Inclusion criteria: (1) aged ≥50 years; (2) residence in the study area for more than 1 year; (3) existing cognitive complaint by the patient himself/herself or family representative; (4) existing vascular factors, such as cerebral hemorrhage, cerebral infarction, leukoaraiosis, hypertension, diabetes, hyperlipidemia, and coronary artery disease.

Exclusion criteria: (1) diagnosed with dementia previously; (2) unconscious or with mental disorders or severe somatic diseases affecting neuropsychological assessment; (3) unable or unwilling to attend the site visit due to other reasons.

### 3.2 The definition of VaMCI

According to the 2019 Chinese Guideline for the Diagnosis and Treatment of VCI (Zhang & Jia, 2019), the definition of VaMCI: (1) existing chief complaint of cognitive decline by himself/herself or family representative, and existing evidence of cognitive decline by cognitive function assessment; (2) existing history of stroke such as cerebral hemorrhage, cerebral infarction, or vascular risk factors, such as leukoaraiosis, hypertension, diabetes, hyperlipidemia, and coronary heart disease; (3) the Montreal Cognitive Assessment (MoCA) scale scored <26, Clinical Dementia Rating scored 0 or 0.5, and Activity of Daily Living scale (ADL) scored ≤26; (4) clarification of the predominance of vascular factors in cognitive impairment; (5) excluding cognitive impairment caused by other nonvascular factors.

### 3.3 Cognitive function assessment

Cognitive function was assessed with MoCA (Beijing version). The assessment took 10–15 mins. The abilities of visual space, executive and naming, memory, attention, language, abstraction, and orientation were assessed. If the duration of education was less than 12 years, 1 point was added. The total score was 30 points. The MoCA score <26 was considered cognitive impairment (Gao et al., 2014). Through this assessment, participants were divided into VaMCI group and normal cognition (NC) group. Participants would also evaluate the Mini–Mental State Examination (MMSE) scale. In addition, ADL had 20 items, and each item scored 1–4 points. The lower the score, the stronger the ability of daily living. If the score of ADL >26, it meant that the assessed had some difficulties in living independently.

### 3.4 Other data collection

We also collected basic information, such as date of birth and sex. The marital status of the patients was mainly divided into married, divorced/widowed. The smoking status was divided into never smoking, light smoking (cumulative smoking <3600 packages), and heavy smoking (cumulative smoking ≥3600 packages). The drinking status was divided into no drinking, light drinking (<75 g alcohol/week), and heavy drinking (≥75 g alcohol/week). The level of education was divided into three categories: less than 6 years, 6–12 years, and more than 12 years. The exercise status was divided into less exercise (<210 min/week), general exercise (210–420 min/week), and regular exercise (>420 min/week).

### 3.5 Statistical methods

The measurement data were presented as means ± standard deviation, and the enumeration data were presented as frequency and percentage. *T*‐test was used to compare the measurement data such as age and the duration of diabetes between the two groups, and chi‐square test was used to compare the enumeration data, such as sex, education level, and medical history. If *p* < 0.05, the data of the two groups were considered to have statistical differences. Univariate logistic regression analysis was used to analyze the correlation between risk factors (including age, sex, education level, etc.) and VaMCI. According to the results of univariate logistic regression analysis, variables with *p* < 0.05 were selected for correction. Multivariate logistic regression analysis was used to analyze the correlation among diabetes, risk factors, and VaMCI, and the OR, 95% confidence interval, and *p* value were calculated. The above analysis was performed using SPSS 25.0 statistical software.

## 4 RESULTS

A total of 2020 participants were included in the analysis, including 1140 with VaMCI and 880 with NC. There were 515 males in the participants with VaMCI and 416 males in the participants with NC. There were significant statistical differences in the proportion of participants in different age groups between the two groups (*p* < .001) (Table 1). Among VaMCI patients, the proportions of patients aged 50–60, 60–70, 70–80, and >80 were 23.5%, 39.8%, 33.5%, and 3.2%, respectively. At the same time, there were also statistical differences in the proportion of patients with education, smoking, and marital status between the two groups. There was no significant difference in BMI, drinking, physical activity, diabetes duration, or other vascular factors between the two groups. Importantly, the proportion of diabetes between the two groups was statistically different (*p* = 0.025), with 25.5% of patients with diabetes in VaMCI group and 21.3% of patients with diabetes in NC group. However, for the assessment of cognitive function, there were some differences in the data of MMSE and MoCA between the two groups (*p* < 0.001).

**TABLE 1:** Baseline characteristics of the 2020 participants.

		Vascular mild cognitive impairment (*n* = 1140)	Normal cognition (*n* = 880)	*p*
		*n*(%) or mean ± SD	*n*(%) or mean ± SD	
**Sex(male)**		515(45.2%)	406(46.1%)	0.69
**BMI**		24.7 ± 3.2	24.6 ± 3.1	0.39
**Age**				<0.001
	**50–60**	268(23.5%)	301(34.2%)	
	**60–70**	453(39.8%)	336(38.1%)	
	**70–80**	381(33.5%)	228(25.9%)	
	**>80**	37(3.2%)	16(1.8%)	
**Education**				<0.001
	**<6**	214(18.8%)	102(11.6%)	
	**6–12**	655(57.5%)	445(50.5%)	
	**>12**	270(23.7%)	334(37.9%)	
**Marital status**				0.009
	**Married**	1045(91.9%)	833(94.9%)	
	**Divorced/separated/widowed**	92(8.1%)	45(5.1%)	
**Smoking status**				<0.001
	**Never**	853(75.4%)	672(77.2%)	
	**<3600 packages totally**	74(6.5%)	89(10.2%)	
	**≥3600 packages totally**	204(18.0%)	110(12.6%)	
**Alcohol consumption**				0.396
	**None**	896(79.2%)	705(80.8%)	
	**<75 g alcohol/week**	60(5.3%)	59(6.8%)	
	**≥75 g alcohol/week**	175(15.5%)	109(12.5%)	
**Physical activity**				0.22
	**<210**	304(40.9%)	218(37.4%)	
	**210–420**	248(33.4%)	221(37.9%)	
	**>420**	191(25.7%)	144(24.7%)	<0.001
**Diabetes**		290(25.5%)	187(21.3%)	0.025
**Diabetes duration**		8.6 ± 6.4	7.5 ± 5.9	0.06
**Other vascular factors**		1137(99.9%)	880(99.8%)	0.209
**MMSE**		28.2 ± 1.0	29.1 ± 0.9	<0.001
**MoCA**		22.4 ± 2.3	27.3 ± 1.2	<0.001

Abbreviations: MMSE, the Mini–Mental State Examination; MoCA, Montreal Cognitive Assessment; SD, standard deviation.

In univariate logistic regression analysis, VaMCI was used as a dependent variable, and sex, age, BMI, smoking, drinking, education, marital status, exercise, diabetes, the duration of diabetes, and other vascular factors were used as independent variables. The results of the analysis (Table 2) showed there was a positive correlation between age, heavy smoking, and diabetes and VaMCI, whereas there was a negative correlation between married, education (especially high education level), light smoking, and general exercise and VaMCI. However, the duration of diabetes and other vascular factors had no correlation with VaMCI.

**TABLE 2:** Univariate association (OR, and 95% CI) between vascular mild cognitive impairment (VaMCI) and diabetes, other factors using logistic regression models.

	OR	95% CI	*p*
**Sex**	1.04	0.87–1.24	0.70
**Age(years)**	1.03	1.02–1.04	<0.001
**BMI(kg/m^2^)**	1.01	0.98–1.04	0.39
**Smoking(< 3600 packs totally)**	0.66	0.47–0.91	0.01
**Smoking(≥3600 packs total lifetime)**	1.46	1.14–1.88	0.003
**Alcohol(< 3 times/week or <25 g alcohol/time)**	0.80	0.55–1.16	0.24
**Alcohol(≥3 times/week or ≥25 g alcohol/time)**	1.26	0.98–1.64	0.08
**Education(primary or junior secondary)**	0.70	0.54–0.91	0.01
**Education(more than junior high school)**	0.39	0.29–0.51	<0.001
**Marital status(married)**	0.61	0.43–0.89	0.01
**Physical activity (210–420 min/week)**	0.79	0.63–0.99	0.05
**Physical activity (>420 min/week)**	0.90	0.74–1.10	0.32
**Diabetes**	1.27	1.03–1.57	0.03
**Duration of diabetes**	1.03	0.91–1.54	0.07
**Other vascular factors**	1.18	0.91–1.54	0.21

Abbreviations: BMI, body mass index; CI: confidence interval; OR, odds ratio.

In multivariate logistic regression analysis, the correlation between diabetes, multiple risk factors, and VaMCI was corrected. The results showed that only diabetes and education were still associated with VaMCI (Table 3). There was a positive correlation between diabetes and VaMCI (OR = 1.04, 95% CI: 1.01–1.09, *p* = 0.05), and a negative correlation between education and VaMCI (OR = 0.60, 95% CI:0.45–0.81, *p* = 0.001) That was, diabetes might increase the incidence of VaMCI by 4%, and a high level of education would reduce the incidence of VaMCI by 40%. The duration of diabetes and other vascular factors still had no correlation with VaMCI.

**TABLE 3:** Multivariate association (OR, 95% CI, and *p* value) between the vascular mild cognitive impairment (VaMCI) and diabetes, other factors using logistic regression models.

	OR	95% CI	*p*
**Sex**	1.22	0.77–1.92	0.40
**Age**	1.01	0.98–1.04	0.48
**Smoking**	1.08	0.80–1.46	0.60
**Education**	0.60	0.45–0.81	0.001
**Marital status**	0.53	0.23–1.23	0.14
**Diabetes**	1.04	1.01–1.09	0.05
**Duration of diabetes**	0.67	0.25–1.76	0.41

Abbreviations: CI: confidence interval; OR, odds ratio.

## 5 DISCUSSION

From the above results, diabetes was indeed positively correlated with VaMCI, so we hypothesize that diabetes may be a risk factor for VaMCI. The results of an aging cohort study in Singapore were consistent with our study. It found that diabetes increased the risk of mild cognitive impairment (MCI) and increased the risk of MCI progressing to dementia by following 2804 participants for about 3 years (Ng et al., 2016). The Atherosclerosis Risk in Communities (ARIC) Study concluded that diabetes was significantly associated with the progression from NC to MCI but not with the progression from NC or MCI to dementia, following 5099 participants for a median 5 years (Rawlings et al., 2019). A Swedish team followed a population of 2522 cases for 12 years and found that people with poorly controlled glycated hemoglobin had double the risk of progressing from NC to MCI and triple the risk of progressing from MCI to dementia (Dove et al., 2021). The above three studies with different follow‐up periods did not categorize the etiology of MCI, but all of them suggested that the effects of diabetes on cognitive function are long‐term and gradually accumulative. That was, diabetes may be involved in the whole process from NC to MCI to dementia.

The pathological mechanisms by which diabetes affects cognitive function have also been studied. In terms of pathological structure, a team found that diabetic patients had a larger volume of white matter hyperintensity and a faster accumulation of white matter hyperintensity by brain magnetic resonance imaging (Marseglia et al., 2019). White matter hyperintensity represents demyelination and axonal loss and is an important pathological factor in cognitive decline. Diffusion tensor imaging sequence further revealed the presence of microstructural abnormalities in multiple cortical areas, projections, and limbic structures in diabetic patients, which were closely related to cognitive functions, such as memory, information processing, executive function, and attention (Moghaddam et al., 2019). Among patients with suspected VCI, diabetic patients had more severe brain atrophy and more lacunar cerebral infarcts (Groeneveld et al. 2019). In terms of pathological organization, neuroinflammation and oxidative stress due to the activation of astrocyte and microglia could lead to neuronal loss, resulting in cognitive impairment (Liyanagamage & Martinus, 2020; Xiong et al., 2023; Zhang et al., 2023). Insulin resistance in the hippocampal tissue, with amyloid deposition, could also lead to neuronal damage, which could cause cognitive decline (Ertas et al., 2023). In addition, mitochondrial homeostasis imbalance, ferroptosis, and neuronal autophagy may also be potential mechanisms for diabetes‐induced cognitive impairment (Chang et al., 2023; Fakih et al., 2020; Tang et al., 2022; Xie et al., 2023; Zheng et al., 2022).

The duration of diabetes may also influence VaMCI. Therefore, our study analyzed the correlation between diabetes duration and VaMCI. Unfortunately, we did not find an association between diabetes and VaMCI, which may be contrary to some previous related studies. The ARIC study also found that older adults with diabetes duration of more than 5 years had a higher risk of cognitive impairment than those with diabetes duration of less than 5 years (Rawlings et al., 2019). A study from the National Health and Nutrition Examination Survey in China found that the longer the duration of diabetes, the higher the risk of cognitive impairment, mainly in terms of processing speed and attention (Tang et al., 2023). Although all of the above studies found that the longer the duration of diabetes, the higher the risk of developing cognitive impairment, our results were different. The reason may be that the duration of diabetes in our study was retrospective data, which may have recall bias. Therefore, subsequent studies could conduct long‐term follow‐up of diabetic patients in the NC group to analyze the effect of diabetes duration on the incidence of VaMCI.

Education is also an important factor associated with VaMCI. A high level of education may prevent the development of VaMCI. Like our study, several studies from different regions abroad had shown that older adults with high levels of education were less likely to suffer from cognitive decline (Arguvanli et al., 2015; Barba et al., 2021; Peeters et al., 2020). The impact of education on cognitive function in old age was primarily through the promotion of cognitive reserve, which persists from early adulthood into old age (Liu & Lachman, 2020). In addition, the good lifestyle and control of blood glucose, blood pressure, and lipids in people with high levels of education may explain why they are less likely to suffer from cognitive decline.

This study still had several limitations. First, our study was based on cross‐sectional data and could not be followed up to observe longitudinal changes, which was insufficient to deal with the confounding factors between diabetes and VaMCI. Second, in the retrospective data, there may be data bias in terms of the duration of diabetes. Shorter or longer duration of diabetes may affect the results of data analysis. The low rate of diagnosis of diabetes, about half of all people with diabetes were not diagnosed in time, could also affect the accuracy of data analysis. Third, the lack of data on glycemic control, such as glycosylated hemoglobin, the use of hypoglycemic drugs, and so on, made the analysis of the correlation between diabetes and VaMCI incomplete.

In a word, we found that diabetes had a positive correlation with VaMCI, and high education had a negative correlation with VaMCI. Diabetes and education level may indicate the incidence risk of VaMCI. Therefore, early identification and timely intervention of diabetes may reduce the risk of VaMCI and achieve early prevention of VaMCI.

## AUTHOR CONTRIBUTIONS

**Yu Zhang**: Data curation; formal analysis; visualization; writing—original draft; writing—review and editing. **Wenna Liu**: Methodology; project administration; supervision; writing—review and editing. **Chen Fu**: Writing—review and editing. **Xuemei Liu**: Writing—review and editing. **Xiaobing Hou**: Investigation. **Huanmin Niu**: Investigation. **Tao Li**: Investigation. **Chunyan Guo**: Data curation; formal analysis; investigation. **Aixun Li**: Data curation; formal analysis; investigation. **Baoxin Chen**: Conceptualization; funding acquisition; methodology; project administration; writing—review and editing. **Xianglan Jin**: Conceptualization; funding acquisition; methodology; project administration; writing—review and editing.

## CONFLICT OF INTEREST STATEMENT

The authors declare that the research was conducted in the absence of any commercial or financial relationships that could be construed as a potential conflicts of interest.

### PEER REVIEW

The peer review history for this article is available at https://publons.com/publon/10.1002/brb3.3477.

## Data Availability

The original contributions presented in the study are included in the article; further inquiries can be directed to the corresponding author.

## References

[brb33477-bib-0001] Arguvanli, S. , Akin, S. , Deniz Şafak, E. , Mucuk, S. , Öztürk, A. , Mazicioğlu, M. M. , Kizilçay, H. D. , & Göçer, Ş. (2015). Prevalence of cognitive impairment and related risk factors in community‐dwelling elderly in Kayseri, Turkey. Turkish Journal of Medical Sciences, 45, 1167–1172. 10.3906/sag-1406-149 26738363

[brb33477-bib-0002] Barba, C. , Garcia, A. , Clay, O. J. , Wadley, V. G. , Andel, R. , Dávila, A. L. , & Crowe, M. (2021). Quality of education and late‐life cognitive function in a population‐based sample from Puerto Rico. Innovation in Aging, 5, igab016. 10.1093/geroni/igab016 34169152 PMC8219031

[brb33477-bib-0003] Celis‐Morales, C. A. , Franzén, S. , Eeg‐Olofsson, K. , Nauclér, E. , Svensson, A.‐M. , Gudbjornsdottir, S. , Eliasson, B. , & Sattar, N. (2022). Type 2 diabetes, glycemic control, and their association with dementia and its major subtypes: Findings from the Swedish national diabetes register. Diabetes Care, 45(3), 634–641. 10.2337/dc21-0601 35077536

[brb33477-bib-0004] Chang, Y. , Wang, C. , Zhu, J. , Zheng, S. , Sun, S. , Wu, Y. , Jiang, X. , Li, L. , Ma, R. , & Li, G. (2023). SIRT3 ameliorates diabetes‐associated cognitive dysfunction via regulating mitochondria‐associated ER membranes. Journal of Translational Medicine, 21(1), 494. 10.1186/s12967-023-04246-9 37481555 PMC10362714

[brb33477-bib-0005] Dove, A. , Shang, Y. , Xu, W. , Grande, G. , Laukka, E. J. , Fratiglioni, L. , & Marseglia, A. (2021). The impact of diabetes on cognitive impairment and its progression to dementia. Alzheimer's & Dementia, 17(11), 1769–1778. 10.1002/alz.12482 34636485

[brb33477-bib-0006] Ertas, B. , Hazar‐Yavuz, A. N. , Topal, F. , Keles‐Kaya, R. , Karakus, Ö. , Ozcan, G. S. , Taskin, T. , & Cam, M. E. (2023). *Rosa canina* L. improves learning and memory‐associated cognitive impairment by regulating glucose levels and reducing hippocampal insulin resistance in high‐fat diet/streptozotocin‐induced diabetic rats. Journal of Ethnopharmacology, 313, 116541. 10.1016/j.jep.2023.116541 37088237

[brb33477-bib-0007] Fakih, W. , Mroueh, A. , Salah, H. , Eid, A. H. , Obeid, M. , Kobeissy, F. , Darwish, H. , & El‐Yazbi, A. F. (2020). Dysfunctional cerebrovascular tone contributes to cognitive impairment in a non‐obese rat model of prediabetic challenge: Role of suppression of autophagy and modulation by anti‐diabetic drugs. Biochemical Pharmacology, 178, 114041. 10.1016/j.bcp.2020.114041 32439335

[brb33477-bib-0008] Gao, X. , Zou, C. S. , Duan, C. B. , Yu, H. Y. , Qin, B. , Bao, L. , & Gao, F. K. (2014). The application of Montreal cognitive assessment in urban Chinese residents of Beijing with amnestic mild cognitive impairment. Chinese Journal of Neuroimmunology and Neurology, 21(04), 250–253.

[brb33477-bib-0033] Groeneveld, O. N. , Moneti, C. , Heinen, R. , de Bresser, J. , Kuijf, H. J. , Exalto, L. G. , Boomsma, J. M. F. , Kappelle, L. J. , Barkhof, F. , Prins, N. D. , Scheltens, P. , van der Flier, W. M. , Biessels, G. J. , & TRACE‐VCI study group . (2019). The Clinical Phenotype of Vascular Cognitive Impairment in Patients with Type 2 Diabetes Mellitus. Journal of Alzheimer's Disease, 68(1), 311–322. 10.3233/JAD-180914 30775988

[brb33477-bib-0010] Hsiung, G.‐Y. R. , Donald, A. , Grand, J. , Black, S. E. , Bouchard, R. W. , Gauthier, S. G. , Loy‐English, I. , Hogan, D. B. , Kertesz, A. , Rockwood, K. , & Feldman, H. H. (2006). Outcomes of cognitively impaired not demented at 2 years in the Canadian Cohort study of cognitive impairment and related dementias. Dementia and Geriatric Cognitive Disorders, 22, 413–420. 10.1159/000095751 16966831

[brb33477-bib-0011] Iadecola, C. , Duering, M. , Hachinski, V. , Joutel, A. , Pendlebury, S. T. , Schneider, J. A. , & Dichgans, M. (2019). Vascular cognitive impairment and dementia: JACC scientific expert panel. Journal of the American College of Cardiology, 73(25), 3326–3344. 10.1016/j.jacc.2019.04.034 31248555 PMC6719789

[brb33477-bib-0012] Kalaria, R. N. (2017). The pathology and pathophysiology of vascular dementia. Neuropharmacology, 134(Part B), 226–239. 29273521 10.1016/j.neuropharm.2017.12.030

[brb33477-bib-0013] Litkowski, E. M. , Logue, M. W. , Zhang, R. , Charest, B. R. , Lange, E. M. , Hokanson, J. E. , Lynch, J. A. , Vujkovic, M. , Phillips, L. S. , Hauger, R. L. , Lange, L. A. , Raghavan, S. , & Va Million Veteran Program (MVP) . (2023). Mendelian randomization study of diabetes and dementia in the Million Veteran Program. Alzheimer's & Dementia, 19(10), 4367–4376. 10.1002/alz.13373 PMC10592524 37417779

[brb33477-bib-0014] Liu, Y. , & Lachman, M. E. (2020). Education and cognition in middle age and later life: The mediating role of physical and cognitive activity. Journals of Gerontology. Series B, Psychological Sciences and Social Sciences, 75, e93–e104. 10.1093/geronb/gbz020 30955036 PMC7424279

[brb33477-bib-0015] Liyanagamage, D. S. N. K. , & Martinus, R. D. (2020). Role of mitochondrial stress protein HSP60 in diabetes‐induced neuroinflammation. Mediators of Inflammation, 2020, 8073516. 10.1155/2020/8073516<./bib> 32410865 PMC7201845

[brb33477-bib-0016] Marseglia, A. , Fratiglioni, L. , Kalpouzos, G. , Wang, R. , Bäckman, L. , & Xu, W. (2019). Prediabetes and diabetes accelerate cognitive decline and predict microvascular lesions: A population‐based cohort study. Alzheimer's & Dementia, 15(1), 25–33. 10.1016/j.jalz.2018.06.3060 30114414

[brb33477-bib-0017] Ng, T. P. , Feng, L. , Nyunt, M. S. Z. , Feng, L. , Gao, Q. , Lim, M. L. , Collinson, S. L. , Chong, M. S. , Lim, W. S. , Lee, T. S. , Yap, P. , & Yap, K. B. (2016). Metabolic syndrome and the risk of mild cognitive impairment and progression to dementia: Follow‐up of the Singapore longitudinal ageing study cohort. JAMA Neurology, 73(4), 456–463. 10.1001/jamaneurol.2015.4899 26926205

[brb33477-bib-0018] Peeters, G. , Kenny, R. A. , & Lawlor, B. (2020). Late life education and cognitive function in older adults. International Journal of Geriatric Psychiatry, 35, 633–639. 10.1002/gps.5281 32043687

[brb33477-bib-0019] Rawlings, A. M. , Sharrett, A. R. , Albert, M. S. , Coresh, J. , Windham, B. G. , Power, M. C. , Knopman, D. S. , Walker, K. , Burgard, S. , Mosley, T. H. , Gottesman, R. F. , & Selvin, E. (2019). The association of late‐life diabetes status and hyperglycemia with incident mild cognitive impairment and dementia: The ARIC study. Diabetes Care, 42, 1248–1254. 10.2337/dc19-0120 31221696 PMC6609963

[brb33477-bib-0020] Sanjari Moghaddam, H. , Ghazi Sherbaf, F. , & Aarabi, M. H. (2019). Brain microstructural abnormalities in type 2 diabetes mellitus: A systematic review of diffusion tensor imaging studies. Frontiers in Neuroendocrinology, 55, 100782. 10.1016/j.yfrne.2019.100782 31401292

[brb33477-bib-0021] Skrobot, O. A. , Black, S. E. , Chen, C. , Decarli, C. , Erkinjuntti, T. , Ford, G. A. , Kalaria, R. N. , O'brien, J. , Pantoni, L. , Pasquier, F. , Roman, G. C. , Wallin, A. , Sachdev, P. , Skoog, I. , Taragano, F. E. , Kril, J. , Cavalieri, M. , Jellinger, K. A. , Kovacs, G. G. , … & Kehoe, P. G. (2018). Progress toward standardized diagnosis of vascular cognitive impairment: Guidelines from the vascular impairment of cognition classification consensus study. Alzheimer's & Dementia, 14(3), 280–292. 10.1016/j.jalz.2017.09.007 29055812

[brb33477-bib-0022] Tang, W. , Li, Y. , He, S. , Jiang, T. , Wang, N. , Du, M. , Cheng, B. , Gao, W. , Li, Y. , & Wang, Q. (2022). Caveolin‐1 alleviates diabetes‐associated cognitive dysfunction through modulating neuronal ferroptosis‐mediated mitochondrial homeostasis. Antioxidants & Redox Signaling, 37(13–15), 867–886. 10.1089/ars.2021.0233 35350885

[brb33477-bib-0023] Tang, X. , Wang, Y. , Simó, R. , Stehouwer, C. D. A. , & Zhou, J.‐B. (2023). The association between diabetes duration and domain‐specific cognitive impairment: A population‐based study. Journal of Alzheimer's Disease, 91(4), 1435–1446. 10.3233/JAD-220972 36641674

[brb33477-bib-0024] Thomassen, J. Q. , Tolstrup, J. S. , Benn, M. , & Frikke‐Schmidt, R. (2020). Type‐2 diabetes and risk of dementia: Observational and Mendelian randomisation studies in 1 million individuals. Epidemiology and Psychiatric Sciences, 29(29), e118. 10.1017/S2045796020000347 32326995 PMC7214711

[brb33477-bib-0025] Wang, Y. , Li, C. , Liang, J. , Gao, D. , Pan, Y. , Zhang, W. , Zhang, Y. , Zheng, F. , & Xie, W. (2023). Onset age of diabetes and incident dementia: A prospective cohort study. Journal of Affective Disorders, 329, 493–499. 10.1016/j.jad.2023.02.138 36868384

[brb33477-bib-0026] Wolters, F. J. , & Ikram, M. A. (2019). Epidemiology of vascular dementia. Arteriosclerosis, Thrombosis, and Vascular Biology, 39(8), 1542–1549. 10.1161/ATVBAHA.119.311908 31294622

[brb33477-bib-0027] Xie, Z. , Wang, X. , Luo, X. , Yan, J. , Zhang, J. , Sun, R. , Luo, A. , & Li, S. (2023). Activated AMPK mitigates diabetes‐related cognitive dysfunction by inhibiting hippocampal ferroptosis. Biochemical Pharmacology, 207, 115374. 10.1016/j.bcp.2022.115374 36502872

[brb33477-bib-0028] Xiong, F. , Gong, K. , Xu, H. , Tu, Y. , Lu, J. , Zhou, Y. , He, W. , Li, W. , Li, C. , Zhao, L. , Gao, H. , & Zheng, H. (2023). Optimized integration of metabolomics and lipidomics reveals brain region‐specific changes of oxidative stress and neuroinflammation in type 1 diabetic mice with cognitive decline. Journal of Advanced Research, 43, 233–245. 10.1016/j.jare.2022.02.011 36585111 PMC9811331

[brb33477-bib-0029] Zhang, J. J. , Jia, J. P. , & Specialized Committee On Cognitive Disorders Of The Neurology Branch Of The Chinese Physicians Association . (2019). 2019 Chinese guidelines for the diagnosis and treatment of vascular cognitive impairment. National Medical Journal of China, 99(35), 2737–2744.

[brb33477-bib-0030] Zhang, Z.‐T. , Deng, S.‐M. , Chen, C. , He, Q.‐H. , Peng, X.‐W. , Liang, Q.‐F. , Zhuang, G.‐D. , Wang, S.‐M. , & Tang, D. (2023). Pterostilbene could alleviate diabetic cognitive impairment by suppressing TLR4/NF‐кB pathway through microbiota‐gut‐brain axis. Phytotherapy Research, 37(8), 3522–3542. 10.1002/ptr.7827 37037513

[brb33477-bib-0031] Zheng, J. , Wang, Y. , Liu, Y. , Han, S. , Zhang, Y. , Luo, Y. , Yan, Y. , Li, J. , & Zhao, L. (2022). cPKCγ deficiency exacerbates autophagy impairment and hyperphosphorylated tau buildup through the AMPK/mTOR pathway in mice with type 1 diabetes mellitus. Neuroscience Bulletin, 38(10), 1153–1169. 10.1007/s12264-022-00863-4 35596894 PMC9554100

